# Diagnostic accuracy of fecal calprotectin in assessing the severity of inflammatory bowel disease: From laboratory to clinic

**DOI:** 10.22088/cjim.8.3.178

**Published:** 2017

**Authors:** Soheila Moein, Durdi Qujeq, Mostafa Vaghari Tabari, Mehrdad Kashifard, Karimollah Hajian-tilaki

**Affiliations:** 1 Molecular Medicine Research Center, Hormozgan University of Medical Sciences, Bandar Abbas, Iran.; 2 Department of Biochemistry, Faculty of Medicine, Hormozgan University of Medical Sciences, Bandar Abbas, Iran.; 3 Cellular and Molecular Biology Research Center, Health Research Institute, Babol University of Medical Sciences, Babol, Iran.; 4 Department of Clinical Biochemistry, Babol University of Medical Sciences, Babol, Iran.; 5 Cancer Research Cancer, Health Research Institute, Babol University of Medical Sciences, Babol, Iran.; 6 Department of Internal Medicine, Ayatollah Rouhani Hospital, Babol University of Medical Sciences, Babol, Iran.; 7 Social Determinant of Health Research Center, Health Research Institute, Babol University of Medical Sciences, Babol,Iran.; 8 Department of Biostatistics and Epidemiology, Babol University of Medical Sciences, Babol, Iran.

**Keywords:** Calprotectin, Inflammatory bowel disease, Marker, Severity

## Abstract

**Background:**

Inflammatory bowel disease (IBD) involves chronic inflammation of the digestive tract. In the past decades, fecal calprotectin has been proposed as a useful biomarker for the differential diagnosis between IBD patients and healthy controls. We designed this study to evaluate the diagnostic ability of fecal calprotectin (FC) and conventional inflammatory markers in IBD patients.

**Methods:**

Thirty patients who underwent colonoscopy were cases and thirty *healthy subjects undergoing colonoscopy* as part of a medical check-up were the controls. These 2 *groups were* evaluated with regard to *age* and* sex*. Severity of the disease was evaluated based on disease endoscopic index. FC, Cross reactive protein (CRP) and Erythrocyte sedimentation rate (ESR) were measured using ELISA, colorimetric and Westergren methods, respectively. The obtained data were analyzed by independent test, correlation test and receiver operating characteristic (ROC) curve analysis. A p<0.05 was considered statistically significant.

**Results:**

Fecal calprotectin level in the case group increased compared to control group (p<0.05). Moreover FC has stronger correlation with disease endoscopic activity than conventional inflammatory markers (r=0.847 versus r= -0.44 for CRP and r=0.054 for ESR in Crohn's disease and r=0.798 versus r=0.463 for CRP and r=0.467 for ESR in ulcerative colitis). Receiver operating characteristic (ROC) curve analysis showed FC has larger area under the curve (AUC) than conventional inflammatory markers (1 versus 0.849 for CRP and 0.846 for ESR).

**Conclusion:**

Discriminating IBD patients from healthy controls was better for FC than conventional inflammatory markers. Additionally, the results produced by FC correlate with the severity of IBD.

M-It is a well-established concept that IBD is a chronic inflammatory disease which contains two types of chronic intestinal disorders: Crohn's disease (CD) and ulcerative colitis (UC) (1). There is accumulating evidence that over the past three decades, the prevalence of IBD in western countries and in developing countries has increased significantly (2). Alterations of minerals and trace element metabolism can be induced by IBD (3). Previous work has indicated that colonoscopy and histopathological examination on tissue biopsy samples are the gold standard for diagnosing IBD and monitoring patients with this disease through these methods are invasive and expensive (4). In addition, access to the affected areas is difficult in CD (5).

Earlier studies have suggested that the laboratory methods most commonly used in the diagnosis of IBD are CRP and ESR. As reported by many investigators, these methods do not have adequate sensitivity and specificity (6, 7). Many studies have been conducted to find a specific marker for diagnosing and monitoring IBD. In many studies, FC has been known as the best noninvasive specific marker in this regard (6). 

Clearly, calprotectin is a calcium binding protein which belongs to the S-100 protein family. It has an essential role in inflammatory process, regulates myeloid cell adhesion to endothelium and extracellular matrix, and furthermore, it has antimicrobial role by competition for zinc through zinc chelation ability. As previously demonstrated, calprotectin comprises the majority of neutrophil cytosol soluble protein content. During the inflammatory process, calprotectin is released due to the degranulation of neutrophil granulocytes, so an increased FC level is the direct consequence of neutrophil degranulation due to mucosal damage which occurs in inflammatory disease (8, 9). Some investigators suggest that the measurement of FC is a useful tool to accurately distinguish IBD from non-inflammatory bowel diseases, such as Irritable bowel syndrome (IBS) (10). Moreover, some researchers have reported that FC can predict disease recurrence, especially in the case of UC (5). 

There is evidence that the assessment of the disease activity is an important factor for evaluating the response to treatment and patient monitoring. There are reports in the literature showing that a number of scoring systems for the assessment of endoscopic activity in IBD patients have been developed. Ulcerative colitis endoscopic index of severity (UCEIS) and simple endoscopic scoring in crohn’s diseases (SES-CD) are the most widely-used among them. Preliminary studies revealed that UCEIS score represents a validated UC endescopic index and SES-CD score represents a validated index to *assess* CD severity. 

It is important to note that UCEIS is a newly-developed scoring system which was reported by Travis et al. in 2012. It is already well known that the score of UCEIS consists of vascular pattern, bleeding, erosions and ulcers ranging from 3 (remission) to 11 (severe) according to previous published data (11). Latest reports demonstrated that SES-CD was developed by Daperno et al. in 2004. It is important to emphasize that this system has four variables including: size of ulcers, ulcerated surface, affected surface and presence of narrowing according to previously reported data (12). It is generally accepted that SES-CD ranges from 0 to 56 (0-2) defined as remission, 3 to 6 mild, 7 to 15 moderate and ≥16 severe (13). The aim of this study was to compare FC and conventional inflammatory markers utility in the discrimination between IBD patients and healthy controls and assessment of the severity of the disease in IBD patients.

## Methods

This Study was carried out in North of Iran on patients admitted to Ayatollah Rouhani Hospital Endoscopy Department during 2014-2016. A complete clinical history was taken from patients undergoing colonoscopy and was asked to provide a stool sample before consuming the prescribed drugs for colonoscopy preparation. Additionally, fasting blood samples were taken from the patients before colonoscopy, serum samples were separated by centrifugation at 3000 rpm for 10 minutes at room temperature. Colonoscopy was performed up to cecum after propofol injection. 

In some patients, colonoscopy was performed up to the terminal ileum, furthermore, biopsy was taken from inflamed mucosa in new cases for histopathological examination and confirmed IBD diagnosis. Based on the colonoscopgic findings and histopathological reports and also consultation with a gastroenterologist and a biostatistician, of the 42 IBD diagnosed patients, 12 patients were excluded from the study because of rejected IBD diagnosis in histopathological examination, in addition to other systemic conditions along with IBD and regular use of NSAIDs. Around 30 patients (14 females and 16 males) with the mean age of 31.0±7 year were considered as the patient group. Among the 30 IBD patients, 8 patients had Crohn's disease while 22 of them had ulcerative colitis. Thirty healthy subjects (14 females and 16 males) with normal colonoscopys and mean age of 32.0±7 year were selected as the control group. All incident IBD cases diagnosed with clinical and colonoscopic findings and confirmed by histopathological examination was one of the inclusion criteria. While the exclusion criteria were: (a) insufficient stool sample provided (b) more than 1month delay between the FC sample and the endoscopy date (c) previous upper or lower GI endoscopy (d) the use of NSAID 1 month before stool sample preparation and (e) any systemic disorders along with IBD. 

Informed consent was obtained from all patients for the subsequent use of their blood and stool samples. The present study conformed to the World Medical Association Declaration of Helsinki (13). Disease severity was evaluated based on SES-CD and UCEIS. Fecal calprotectin was measured by ELISA sandwich test according to kit’s instructions (Buhlmann Co, Switzerland, cat number .EK-CAL), the kit suggested a cut-off level of <50µg/g. CRP was measured by Auto Analyzer (Hitachi Co. Japan) according to kit’s instruction manual (Bionic Co. Iran) and ESR was measured by Westergren method. Descriptive statistics and analysis were performed in SPSS Version 20 and independent t-test and p-values less than 0.05 were considered statically significant. Pearson correlation coefficient was used to determine the correlation between variables. We also used receiver operating characteristic (ROC) analysis to estimate the accuracy index of CRP, ESR and fecal calprotectin in predicting IBD diagnosis. The area under the curve (AUC) as diagnostic accuracy and its 95% confidence interval (CI) was calculated. 

## Results

The findings of this study showed that the average of FC level, CRP and ESR in IBD patients is higher than the healthy controls (table1). 

**Table 1:** Comparison of means of CRP, ESR and fecal calprotectin between IBD patients and control subjects

**Outcome variables**	**Case(n=30)** **Mean±SD**	**Control(n=30)** **Mean±SD**	**pvalue**
Age (year)	31.0±7.0	32.0±7.0	0.509
CRP (mg/l)	13.6±11.0	5.2±2.0	0.001
ESR(mm/h)	42.0±22.0	14.0±8.0	0.001
Fecal calprotectin (µg/g)	482.2±265.0	28.1±11.5	0.001

Data are presented as mean±SD.

There was no significant difference in the average of CRP and ESR levels between the two groups of IBD patients (CD and UF). The average of FC level is higher in patients with Crohn's disease than UC patients, but this difference is not statistically significant (table 2). The average of disease endoscopic activity index score for crohn's disease (SES-CD) and ulcerative Colitis patients (UCEIS) was 11.0±8.1 and 6.86±1.95 (mean±SD), respectively. FC has significant correlation with SES-CD and UCEIS (Pearson correlation coefficient (r) between FC and SES-CD was 0.847 and between FC and UCEIS was 0.798 p<0.05) but CRP and ESR levels have significant correlation only with UCEIS. FC correlates more closely with UCEIS than the conventional inflammatory markers (CRP, ESR) table 3.

**Table 2:** Comparison of mean±SD of CRP, ESR and fecal calprotectin between two groups of IBD patients

**Outcome variable**	**Crohn** **'s ** **disease**	**Ulcerative Colitis**	**Pvalue**
CRP(mg/l)	15.7±12.4	12.8±10.7	0.540
ESR(mm/h)	46.00±23.00	41.00±23.00	0.642
Fecal calprotectin (µg/g)	619.3±296.3	432.4±240.8	0.088

Data are presented as mean ± SD.

**Table 3:** Correlations between disease endoscopic activity index, conventional inflammatory markers and fecal calprotectin

**Variables**	**CRP**		**ESR**		**Fecal calprotectin**	
	**r**	**Pvalue**	**r**	**Pvalue**	**r**	**Pvalue**
SES-CD* (n=8)	-0.044	0.918	0.054	0.898	0.847	0.008
UCEIS** (n=22)	0.463	0.030	0.467	0.028	0.798	0.001

r= Pearson correlation coefficient

* Simple endoscopic scoring in Crohn’s disease

** Ulcerative colitis endoscopic index of severity

The predictive accuracy of CRP, ESR and FC to distinguish between IBD and non-IBD patients was shown in table 4.The AUC of FC is 1.000, while the AUC of CRP and ESR were 0.849 and 0.846, respectively (table 4). 

**Table 4:** Predictive accuracy of CRP, ESR and FC for diagnosis of IBD

**Variables**	**AUC**	**(95% CI)**	**P-value**
CRP	0.849	0.754,944	<0.001
ESR,1h	0.846	0.742, 0.950	<0.001
FC	1.00	(-)	<0.001

AUC, area under ROC curve; CI, confidence interval

**Figure 1:**
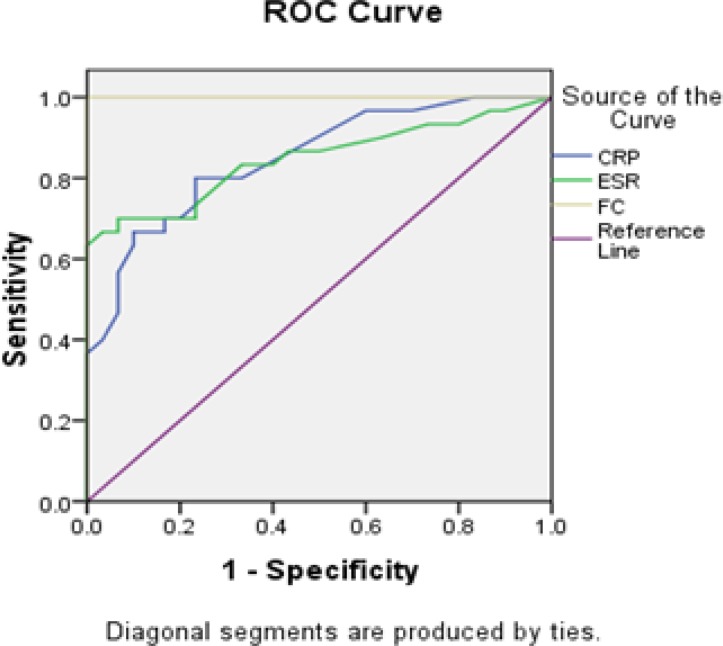
Receiver operating characteristic analysis of CRP, ESR 1h and F*C*.

These results show that the accuracy of FC test in separating subjects with IBD from those without IBD is more than CRP and ESR as a consequence FC test is perfect in differentiating between the subjects with IBD from those without IBD (figure1). The present study suggested 78.4 µg/g as cut-off level for FC by 100% sensitivity and 100% specificity in discriminating IBD from non-IBD patients.

## Discussion

The results of this study indicated that the FC level in patients with IBD is significantly higher compared to control group. These results are consistent with many studies (14-19) but our data showed higher average level of FC in IBD patients compared to other studies (17-19). This difference may be due to population distribution because the majority of current studies on IBD patients are in active phase of the disease and newly diagnosed. Besides we had a low sample size, and our data demonstrated that the patients with CD had elevated FC level compared to patients with UC (table 2). Meanwhile some previous studies do not show any difference in FC levels between CD and UC (4, 15) in which this inconsistency maybe due to the low sample size in our study. The findings of the current study also showed direct correlation between FC level and disease endoscopic activity indexes which is similar to Schoepfer A et al. and Vieira A et al.s studies (17, 18). Schoepfer A et al. showed that FC is more related to SES-CD index than conventional inflammatory markers (19), which is similar to our result, but the finding of this study showed that conventional inflammatory markers (CRP and ESR) have no significant correlation with disease endoscopic activity index in the case of CD (table 3). This could be due to the low number of patients, in addition their weak correlations with UCEIS are in comparison with FC. These results demonstrated that FC has stronger correlation with disease endoscopic activity index than conventional inflammatory marker. Thus FC has better utility in disease endoscopic activity reflection than conventional inflammatory markers. Additionally, our ROC curve analysis showed that fecal calprotectin test has larger area under the curve (AUC) compared to CRP and ESR (table 4 and figure1). As a result, discriminating IBD patients from healthy controls was better for FC than conventional inflammatory markers.

This finding is similar to other studies ( 20 ), but in the current study, AUC for FC was: 1 (table 4), this may be due to the low sample size and population distribution as mentioned above. However, FC is a better laboratory test to distinguish between IBD and healthy controls compared to conventional inflammatory. Consequently, based on our present findings, parallel with previous results, we suggest that it can be used as a useful and non-invasive biomarker for discrimination of IBD patients from healthy controls and also in the assessment of endoscopic activity of IBD. The current study also suggested 78.4 µg/g as the cut-off level for FC by 100% sensitivity and 100% specificity in the discrimination of IBD from non-IBD patients. Yet it should be noted that by increasing the sample size and changing population distribution, the sensitivity and specificity will possibly reduce. In this study, we used SES-CD and UCEI indexes for assessing disease endoscopic activity known as the most accurate index. Moreover, a study on a larger population with other endoscopic index is effective and will help to further clarify the issue. Likewise, further studies are needed to clear the effect of FC measurement on colonoscopy rates. While limiting the clinical usefulness of this test beyond all these positive results and suggestions, some limitations and methodological flaws of our study should be mentioned. Our small sample size might have led to the loss of the power of statistical analysis. The effectiveness of FC test needs to be tested in a much larger population and characterized it as a function of known duration of the IBD. 

In conclusion our finding demonstrated that FC has better effect in the differentiation between the subjects with IBD from those without IBD than conventional inflammatory marker. Our results showed that FC correlates more closely with disease endoscopic activity index than conventional inflammatory markers.
